# Supplementary material for: Expected global suitability of coffee, cashew and avocado due to climate change
Source: PLoS One. 2022 Jan 26;17(1):e0261976. doi: 10.1371/journal.pone.0261976 (PMC8791496; doi:10.1371/journal.pone.0261976)
Supplement: Attachment — Submitted filename: Review1-Emmanuel Junior Zuza.docx [file pone.0261976.s001.docx]

**Review**

This study aimed to assess the current and future suitability of coffee, cashew and avocado production on a global and county scale using biophysical parameters. This study is important because it considers land, soil and climatic predictors that influence the production of the three perennial crops. The authors utilized the GIS-based decision support system CONSUS model for their analysis. However, the paper is missing information on the importance and influence of biophysical factors on the crops current and future suitability. This is vital based on the urgument that such parameters have not been used in modelling works for these crops, hence filling the scientific gap.

Major comments

- There is a need to revise the submission taking into account grammatical errors in the manuscript.
- Authors need to revise their methodology and should make sure that it is clear on what they did. They need to highlight if they used known areas of occurrence of the crops, i.e. presence data. The authors mention a threshold that was set in the methodology but does not show what it was. Ideally, the authors need to make known what this is, i.e. 0,1 with the ranges (suitable, unsuitable, marginal etc.). If you could explain the maximum limiting factor (MLF) and why you used it.
- One of the biggest things when using bioclimatic data from WorldClim is multicollinearity. Would the authors provide a sentence that proves that the bioclimatic variables they used were not correlated, as this has been shown to cause model overfitting?
- The authors should consider breaking the results into different subheadings, i.e. factors determining suitability, the current suitability (global and country-specific) and future suitability (global and country-specific). As it stands, it becomes confusing what the authors are trying to convey.
- The results highlight that cashew has the largest suitable growing areas globally than avocado and coffee under the current climate conditions. It will be more meaningful if you could provide the percentages (%) of these. We can see these on the maps but perhaps consider having a table/fig in the supplemental materials showing the differences based on the classes.
- In the results, there is a mixture of words that make it difficult to understand what you mean, i.e. the minimum temperature of the coldest month and minimum temperature in the coldest season. The authors need to be consistent with the wording.
- The results in table 3 should be presented per crop and not as a whole thing. It makes the reader move back and forth to see what the authors say in the country-specific suitability.
- The authors need to revise their discussion comparing the current crop suitability with the main producing regions. It would make more sense if they separated the two, i.e. discuss the current crop suitability results from those with the main producing areas as they did in the results.
- The authors need to focus on discussing the findings of their study rather than existing literature. For instance, in avocado's discussion from Line 474-479, the authors highlight what others have done. It is recommended to start discussing your results and later compare these to what others have done. This way, the reader understands your findings.

Minor comments

Line 1-2: Consider rewording the title "Expected global suitability of coffee, cashew and avocado due to climate change". Do not use the species name in the title.

Line 12: Replace the word Coffee arabica with coffee. This applies to the rest of the document.

Line 15: replace "or" with "and".

Line 18-19: Restructure your sentence to make more sense. "*We used climate outputs from 14 global circulation models based on three emission scenarios to model the future climate changes impacts on the crops both globally and in main producing countries".*

Line 27-29: Provide how much expansion you are talking about globally, i.e. the percentage (%) increase and the % decrease within the countries (cumulative).

Line 35-36: Include the scientific names of coffee, cashew and avocado. A reference would be great as well.

Line 36-38: Consider rephrasing this sentence.

Line 76-78: Remove the scientific names and include these in the first paragraph of the introduction.

Line 158-159: This is a repetition of what you have already shown. Consider rephrasing this.

Line 228-229: Should be N and S boundaries of Africa, remove "in".

Line 253-256: Consider putting Table 3 in landscape mode as it is not visible in portrait.

Line 284: Low soil pH shall mean by how much? Please consider putting the pH number on there.

Line 290: Best write 2-13% (see Table 3).

Line 311: I think the authors mean cashew suitability and not agricultural suitability. If this is the case, please rephrase the sentence.

Line 318: Check the spelling of Vietnam.

Line 328: Consider revising the sentence.

Line 334-337: Rephrase. The sentence is unreadable.

Line 372-373: Consider revising this sentence as it does not make sense. The authors talk about climate suitability restricting avocado suitability. I think you mean weather parameters, not climate suitability per se.

Line 392-394: Rewrite the sentence.

Line 426-427: The author's highlight, "*Similar trends were found in general".* Which trends are these? It would be important to start highlighting whether climate change will impact coffee production +ve or -ve; thus, the readers know which trends these are. Possibly use the sentence that talks about the reductions first and then highlights what others have done.

Line 448-450: Could you discuss the implications or the importance of considering land and soil requirements for coffee suitability. This will add more scientific knowledge on why modelling studies need to consider these parameters.

Line 506-509. The authors need to reconsider these sentences as they have also utilized bioclimatic data provided by WorldClim. Best highlighting that other studies lack the aspect of biophysical parameters?

Line 527-531: This sentence shows that climatic factors are the most crucial limiting factors for crop production. Basing on this, what could be the best recommendation for the future of crop modelling activities? Could a combination of climatic and soil/land parameters improve the model accuracy?
