# Supplementary material for: Expected global suitability of coffee, cashew and avocado due to climate change
Source: PLoS One. 2022 Jan 26;17(1):e0261976. doi: 10.1371/journal.pone.0261976 (PMC8791496; doi:10.1371/journal.pone.0261976)
Supplement: Attachment — Submitted filename: Reviewers comments - Copy.docx [file pone.0261976.s003.docx]

**Reviewers comments**

The authors have managed to address our concerns and now the article is great. It can be **Accepted** for publication.

Minors.

Line 17: Remove “confirming previous findings”.

Line 45: I think what you mean here is that Coffee arabica is highly sensitive to and dependent on weather variables not climate change per se?

Line 195: Remove “still” from the sentence.

Line 232: Climate suitability or climate factors? Please check this to avoid confusing the reader.

Line 233: Check “in Africa or East Africa” revise this.
